# The immunotherapeutic role of indoleamine 2,3‐dioxygenase in head and neck squamous cell carcinoma: A systematic review

**DOI:** 10.1111/coa.13794

**Published:** 2021-05-30

**Authors:** Daniel J. Lin, James C. K. Ng, Lei Huang, Max Robinson, James O’Hara, Janet A. Wilson, Andrew L. Mellor

**Affiliations:** ^1^ Translational and Clinical Research Institute Newcastle University Newcastle upon Tyne UK; ^2^ ENT Department Freeman Hospital High Heaton, Newcastle upon Tyne UK; ^3^ Centre for Oral Health Research Newcastle University Newcastle upon Tyne UK; ^4^ Institute of Health & Society Newcastle University Newcastle upon Tyne UK

**Keywords:** biomarkers, immune system, immunotherapy, indoleamine‐pyrrole 2,3‐dioxygenase, squamous cell carcinoma of head and neck, tryptophan, tumour microenvironment

## Abstract

**Background:**

Novel cancer immunotherapy seeks to harness the body's own immune system and tip the balance in favour of antitumour activity. The intracellular enzyme indoleamine 2,3‐dioxygenase (IDO) is a critical regulator of the tumour microenvironment (TME) via tryptophan metabolism. The potential immunotherapeutic role of IDO in head and neck squamous cell carcinoma (HNSCC) requires further exploration. We aim to assess the evidence on IDO in HNSCC.

**Methods:**

A systematic review of literature and clinical trials databases.

**Results:**

We included 40 studies: seven involved cell lines: eight assessed tumour immunohistochemistry: ten measured IDO gene transcription: 15 reported on clinical trials. Increased cell line IDO expression was postulated to adversely affect tumour metabolism and apoptosis. Immunohistochemical IDO expression correlated with worse survival. Gene transcription studies associated IDO with positive PD‐L1 and human papillomavirus (HPV) status. Phase I/II clinical trials showed (a) overall response (34%‐55%) and disease control rates (62%‐70%) for IDO1 inhibitor in combination with a PD‐1 inhibitor, (b) similar safety profiles when both are used in combination therapy compared to each as monotherapies and (c) IDO gene expression as a predictive biomarker for response to PD‐L1 therapy.

**Conclusions:**

IDO expression is increased in the TME of HNSCC, which correlates with poor prognosis. However, the exact mechanism of IDO‐driven immune modulation in the TME is an enigma. Future translational studies should map IDO activity during HNSCC treatment and elucidate its precise role in the TME, such research will underpin the development of clinical trials establishing the efficacy of IDO inhibitors in HNSCC.

**Keypoints**

- IDO is integral to TME immunity in HNSCC particularly in HPV‐positive cancers.
- IDO can be used to modulate existing therapies and has applications in combinatorial immunotherapy.
- Retrospective studies have shown its presence in the TME and suggest a link to HNSCC treatment outcome.
- However, the exact mechanism of IDO‐driven immune modulation in the HNSCC TME remains unclear.
- We now require prospective longitudinal studies to track IDO activity and expression throughout HNSCC treatment, thence optimise IDO‐based immunotherapy.

## 1 INTRODUCTION

The burgeoning field of cancer immunotherapy has made significant progress with the application of immune checkpoint inhibitors in the treatment of solid tumours at different sites across the body. Such therapeutic strategies seek to harness the body's own immune system and tip the balance in favour of antitumour immunity. The first clinically validated immune checkpoint therapy targeting cytotoxic T‐lymphocyte‐associated antigen (CTLA‐4) mediated tumour regression and increased overall survival in melanoma patients, but was associated with frequent immune‐related adverse events.^1^, ^2^, ^3^, ^4^, ^5^ Programmed death 1 (PD‐1) protein and its ligand PD‐L1 was subsequently discovered^6^, ^7^ and shown to have good safety and efficacy in inducing durable tumour regression and prolonged stable disease in patients with advanced cancers including non‐small cell lung cancer (NSCLC), melanoma, renal cell, ovarian, colorectal, pancreatic, gastric and breast cancer.^8^ However, the vast majority of head and neck cancer patients, about 80%, remain unresponsive to immune checkpoint inhibitor therapy, highlighting the need for more effective immunotherapies and predictive biomarkers.^9^ IDO1 inhibitors for melanoma, glioblastoma, NSCLC, pancreatic and breast cancer are under investigation by pharmaceutical companies and sponsors.^10^ To date, IDO inhibitors for head and neck cancer have been tested in only several published clinical studies.^11^, ^12^, ^13^, ^14^, ^15^, ^16^, ^17^, ^18^

Head and neck squamous cell carcinoma (HNSCC) is the sixth leading cause of cancer worldwide and is diagnosed in 8000 new patients annually in the UK.^19^ HNSCCs are divided into two clinically, genomically and immunologically distinct subgroups based on their association with human papillomavirus (HPV) infection: (a) the majority of HNSCCs are HPV‐negative and tend to present in older patients, usually with a history of smoking and alcohol use; their tumours are often characterised by p53 mutations and have poor 5‐year survival ranging from 33.8% to 66.8% depending on subsite, whilst (b) HPV‐positive HNSCCs arise mainly in younger, Caucasian, non‐smokers and their tumours are characterised by integration of viral genome and the expression of E6 and E7 viral oncoproteins which result in the inactivation of p53 and retinoblastoma (Rb) protein, and subsequent overexpression of p16, but better prognosis and overall survival as their tumours are often radiosensitive.^20^ Current surgical and non‐surgical treatments for HNSCC have devastating functional and cosmetic consequences. Survival has improved little in the past four decades, that is most less than 50%.^21^ The head and neck tumour microenvironment (TME) is a site of intense immunological activity, driving a recent emergence in immunotherapy being applied to HNSCC.

Indoleamine 2,3‐dioxygenase (IDO) is an intracellular enzyme which plays a critical role in the immunity of the TME via tryptophan metabolism. Its activity is increased in the TME of many cancers and its expression was found to be a negative prognostic indicator in melanoma,^22^ ovarian,^23^ colorectal^24^ and lung cancer.^25^, ^26^ IDO inhibits natural and therapy‐induced antitumour immunity as it catabolises the amino acid tryptophan to generate kynurenine and other immunosuppressive catabolites which activate Foxp3 regulatory T cells and attenuate effector T‐cell responses to inhibit immune‐mediated killing of tumour cells. Due to its important role in TME immunity, IDO is an immune checkpoint which can be potentially exploited to improve treatment outcomes. However, the immunotherapeutic role of IDO in HNSCC requires further exploration.

Our review aims to systematically assess the current literature for pre‐clinical and clinical evidence on the immunotherapeutic role of IDO in HNSCC. Our objectives were to (a) identify all studies which investigated IDO in HNSCC, (b) identify studies which investigated IDO activity and/or expression in HNSCC, (c) evaluate the effectiveness of IDO inhibitors at improving the outcomes of patients with HNSCC, (d) compare the use of IDO inhibitors alone and in combination with other treatments for HNSCC and (e) evaluate the potential for immunotherapeutic strategies involving the IDO pathway in the treatment of HNSCC.

## 2 MATERIALS AND METHODS

### 2.1 Data sources and literature search

A systematic literature search was conducted in Ovid MEDLINE, Ovid Embase, Scopus, Web of Science, Cochrane library and ClinicalTrials.gov databases from inception until present day. The PRISMA guidelines for study selection were followed.^27^ All studies that evaluated the involvement of IDO in HNSCC were systematically retrieved. The following search terms and strategy was used: (“indoleamine 2,3‐dioxygenase” OR “IDO” OR “IDO1” OR “IDO‐1” OR “IDO2” OR “IDO‐2”) AND (“squamous cell carcinoma” OR “squamous cell cancer” OR “SCC”). The titles and abstracts from the initial search results were screened independently by two authors DJL and JCKN. To ensure inclusion of all studies related to HNSCC, DJL and JCKN manually screened the studies with squamous cell carcinoma to include those involving the head and neck region.

### 2.2 Study selection

In the initial screening, the following criteria were required for inclusion: (a) HNSCC from any head and neck subsite (oral, oropharynx, nasopharynx, larynx and hypopharynx), (b) study of IDO expression or activity, (c) all study types (prospective or retrospective, observational or experimental, pre‐clinical or clinical), (d) published in English language only and (e) original articles and conference abstracts. Duplicates, correspondence, review articles and studies without data on IDO in the context of HNSCC were excluded.

### 2.3 Data extraction and analysis

Following the generation of a list of articles meeting the inclusion criteria, DJL and JCKN each performed an in‐depth review of the studies and extracted data for comparison. Similar studies were grouped together for qualitative analysis.

## 3 RESULTS

### 3.1 Included studies

**FIGURE 1:**
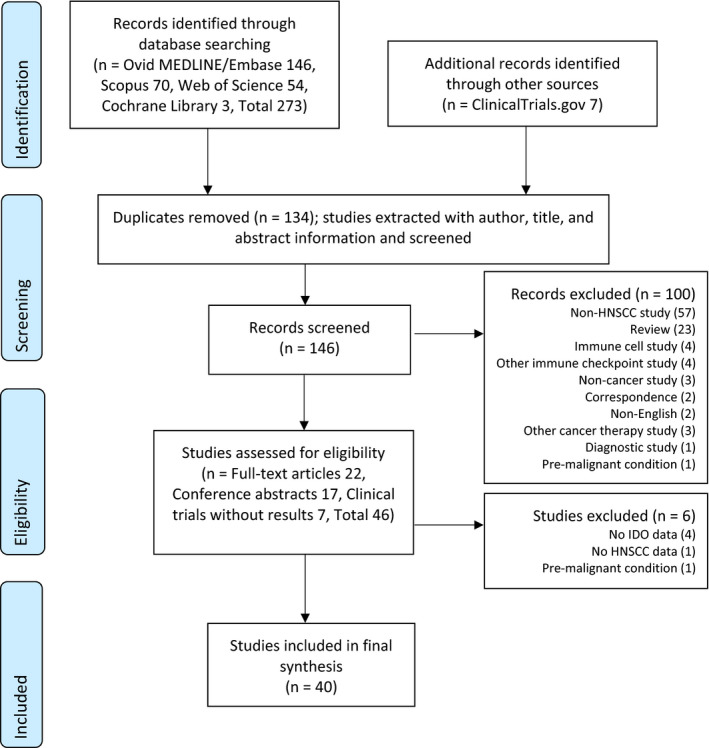
PRISMA flow diagram for study selection. Abbreviations: IDO, indoleamine 2,3‐dioxygenase; HNSCC, head and neck squamous cell carcinoma

A total of 273 studies were identified from databases and seven studies from additional sources, and 146 were screened after removal of 134 duplicates. A total of 100 studies were excluded with reasons described in the PRISMA flow diagram in Figure 1. After full‐text review, 40 studies were included in the final analysis.

Evidence from pre‐clinical and clinical studies involving IDO was extracted. A total of 22 full‐text articles, 17 conference abstracts and seven clinical trials without results were evaluated. Of those seven studies involved cell lines, eight assessed tumour immunohistochemistry (IHC), 10 were IDO gene transcription studies, and 15 others reported on clinical trials (eight published, seven registered without results). All seven cell line studies used different HNSCC cell lines. The prognostic studies involving IHC investigated IDO expression in HNSCC at different subsites, defined as; lower lip, oral cavity, tongue and larynx. The clinical trials compared survival with combination therapies involving IDO1 inhibitor vs monotherapy using PD‐1 inhibitor Pembrolizumab, and assessed IDO expression value as a predictive biomarker for response to PD‐L1 therapy. Additionally, 13 conference abstracts were identified and summarised in Table S1.

### 3.2 Cell line studies on IDO in HNSCC

The seven studies which investigated IDO in HNSCC cell lines, each is an immortalised cell culture developed from a single human HNSCC tumour, are summarised in Table 1. Of those, the majority investigated IDO in oral and oropharyngeal HNSCC cell lines. The studies were heterogeneous in the method of cell line analysis, which ranged from enzyme‐linked immunosorbent assay (ELISA), 2D/3D cell culture, computational simulation model, enzymatic IDO activity assay, quantitative reverse transcriptase polymerase chain reaction (qRT‐PCR), and IFNγ stimulation followed by treatment with cytostatic drugs and quantification of metabolites generated via IDO activity by liquid chromatography tandem mass spectrometry. Additionally, the same cell lines SCC4, SCC15 and SCC25 were only used in 3 of the 7 studies. Bates^28^ showed that SCC15 (T4N1M0) produced significantly more IDO than any other cell line (SCC4, SCC25, UM‐SCC19, UM‐SCC 84, UM‐SCC 92 and UM‐SCC 99). Interestingly in that group, SCC15 was the only cell line derived from T4 stage HNSCC. However, in the computational simulation model^29^ SCC4 had significantly higher IDO expression compared to SCC15 and SCC25. The SCC4 cell line was classified as a non‐responder compared to SCC15 and SCC25 which were classified as responders to PD‐L1 immunotherapy. This characteristic of SCC4 was supported by observations from Liang et al^30^ showing that stimulator of interferon genes (STING) activation significantly induced IDO expression in SCC4. It is also postulated that IDO activity may interfere with tumour metabolism. The studies by Subramanian^31^ and El Jamal^32^ also support the central role of IDO inhibition in upregulation of genes in apoptosis and activation of apoptotic pathways through the suppression of haem oxygenase‐1 and accumulation of reactive oxygen species. Furthermore, Riess et al^33^ used the experimental cyclin‐dependent kinase inhibitor Dinaciclib to suppress IDO activity, thus reducing tryptophan metabolism via the kynurenine pathway in HNSCC cell lines, whilst chemotherapeutics tend to activate this pathway. Chemotherapy may modulate IDO activity indirectly by inducing stressed and dying cells to release damage‐associated molecular patterns (DAMPs), which are sensed to stimulate inflammatory responses. Nevertheless, these findings emphasise the limitations of conventional therapies and the potential of targeted therapies to interfere with HNSCC metabolism. Al‐Samadi et al^34^ showed that applying an IDO1 inhibitor in an in vitro 3D microfluidic chip assay with HSC‐3 induced immune cell migration towards cancer cells. Thus, changing the tumour microenvironment from immunologically “cold” (ie inactive) to “hot” (ie active/inflamed) could enhance the efficacy of other immunotherapeutic drugs in combination.

**TABLE 1:** Cell line studies on IDO in HNSCC

First author, year (country)	Journal	Cell line (subsite)	TNM stage	Assay method	Results interpretation
Riess,^33^ 2020 (Germany)	Frontiers in Immunology	Hypopharyngeal: FADU Pharyngeal: Detroit‐562 Tongue: Cal‐33 PE/CA/PJ‐15 UT‐SCC‐14 UT‐SCC‐15	—	HNSCC cell lines were cultured and treated with IFNγ for 24 h and 72 h, then treated with cytostatic drugs including 5‐fluorouracil (5‐FU), Cisplatin, Gemcitabine and Cetuximab. IDO1 immunofluorescence was performed on the treated cells and kynurenine pathway (KP) metabolites in the cell culture supernatant was quantified by liquid chromatography tandem mass spectrometry	IDO1 expression was low, but inducible upon IFNγ treatment of HNSCC cells. Upon treatment with 5‐FU, Gemcitabine and Cetuximab, IDO1 and additional genes of the KP (*KYAT1, KYAT2* and *KMO*) were induced. Cyclin‐dependent kinase inhibitor Dinaciclib suppressed the KP, whilst conventional chemotherapeutics tend to activate the KP
Al‐Samadi,^34^ 2019 (Finland)	Experimental Cell Research	Oral cavity: tongue: HSC‐3	—	In vitro 3D microfluidic chip assay. HSC‐3 was embedded in human tumour‐derived matrix along with patients’ serum, cancer and immune cells, which were then loaded with anti‐PD‐L1 and IDO1 inhibitors. Immune cell migration and cancer cell proliferation rates were evaluated	IDO1 inhibitor induced immune cell migration towards cancer cells in HSC‐3 and two HNSCC patient samples, which could change the tumour from “cold” to “hot” and enhance the efficacy of other immunotherapeutic drugs in combination. This in vitro 3D microfluidic chip assay could be used to further test immunotherapeutic drugs against patient samples
Bates,^28^ 2018 (USA)	Translational Cancer Research	Oral cavity: tongue: SCC4 SCC15 SCC25 Oropharynx: base of tongue: UM‐SCC19 Oral cavity: UM‐SCC84 Oral cavity: lateral tongue: UM‐SCC92 Oropharynx: UM‐SCC99	T3N0M0 T4N1M0 T2N1 T2N1M0 T2N0M0 T2N0M0 T3N0M0	ELISA to determine the concentration of IDO in cell lysates	SCC15 produced significantly more IDO than any of the six other cell lines. HNSCC cell lines from different hosts can have varying amounts of biomarkers. These differences could be due to the stage of disease, site of tumour, tissue type or genomic differences between patients. These results support personalised medicine in treating HNSCC
Subramanian,^31^ 2018 (USA)	Cancer Research	Cell line not specified	—	HNSCC cell lines grown in 2D culture. Kynurenine levels measured by MS. IDO1 levels in tissue measured by Western Blot	High levels of kynurenine in HNSCC cell lines shown through metabolic profiling via MS. Checkpoint inhibition of IDO1 leads to an upregulation of genes in glycolysis (ACLY, G6PD, COX5A, LPL and PFKL) and apoptosis (CASP7, CASP9, BCL2L11) in vitro
Bates,^29^ 2017 (USA)	Oral Surgery Oral Medicine Oral Pathology Oral Radiology	Oral cavity: tongue: SCC4 SCC15 SCC25	T3N0M0 T4N1M0 T2N1	Cell line‐specific predictive computational simulation models used to predict expression of IDO1	Predicted IDO expression in SCC4 (17.29%), SCC15 (2.75%) and SCC25 (4.97%) with respect to controls. In the simulation model, SSC4 was classified as a non‐responder whilst SCC15 and SCC25 were classed as responders to PD‐L1 immunotherapy
El Jamal,^32^ 2016 (USA)	Cell Division	HNSCC Mouth: CLS‐354 SCC nasal septum: RPMI 2650	—	Enzymatic IDO activity assay, absorbance at 490 nm. Immunoblot with anti‐IDO antibody 1:500 (BioGenes, Berlin, Germany), Western Blot	Described central role of IDO in IFNγ‐induced apoptosis of HNSCC cells by the suppression of HO‐1 leading to the accumulation of ROS and activation of apoptotic pathways
Liang,^30^ 2015 (China)	Biochimica et Biophysica Acta ‐ Molecular Basis of Disease	Oral SCC: HSC‐3 SCC‐4 Normal human keratinocyte: HaCaT	—	IDO mRNA isolation by qRT‐PCR	IDO expression was significantly induced in HaCaT, HSC‐3 and SCC4 by STING activation. Suggests the establishment of HNSCC TME by immunosuppressive cytokines such as IDO could be promoted by 2′‐3′ cGAMP activation of STING

Abbreviations: ELISA, enzyme‐linked immunosorbent assay; TNM, Tumour Node Metastasis; SCC, squamous cell carcinoma; MS, mass spectrometry; HO‐1, haem oxygenase‐1; ROS, reactive oxygen species; STING, stimulator of interferon genes; qRT‐PCR, quantitative reverse transcription polymerase chain reaction; cGAMP, 2′‐5′,3′‐5′cyclic AMP‐GMP.

### 3.3 Tumour immunohistochemistry studies on IDO in HNSCC

The majority of tumour IHC studies were performed on formalin‐fixed paraffin‐embedded tissue blocks. The studies in this group were designed with a retrospective method of analysis; none used prospective, fresh tissue collection or analysis of IDO activity within the tissues. The tissues studied came from the lower lip, oral cavity, tongue, tonsil and larynx. The eight studies included in this group are summarised in Table 2. Anti‐IDO monoclonal antibody clone 10.1 was widely used. IDO staining was most commonly seen at the invasive front of the tumour and increased IDO staining correlated with worse survival.^35^, ^36^, ^37^ Four of the studies^35^, ^36^, ^37^, ^38^ were prognostic studies which reported on IDO expression and correlation with outcome. Only two studies fulfilled all REMARK checklist criteria for prognostic biomarker studies. In a recent prognostic study by Wang et al, an increase in IDO expression was seen in clinical non‐responders to Nimotuzumab (anti‐epidermal growth factor receptor) therapy, suggesting that IDO may be a biomarker of immune status in the TME during therapy in oral SCC patients.^38^

**TABLE 2:** Tumour immunohistochemistry studies on IDO in HNSCC

First author, year (country)	Tumour site/stage	Source/preparation	Primary antibody, manufacturer (clone), dilution	Cases (n)	Method of analysis	Results interpretation	Compliance with REMARK^77^ checklist
Wang,^38^ 2019 (China)	Oral SCC (OSCC)	FFPE tissue sections collected before and after 4‐wk treatment with six cycles of Nimotuzumab	Anti‐IDO1 (Abcam, ab211017), 1:1500	36	Retrospective. IHC slides were assessed by 3 independent pathologists blindly and a staining score was given	Nimotuzumab therapy increased the expression of IDO in the TME of OSCC patients compared with baseline pre‐treatment *P* = .0053, suggesting IDO as a marker of immune status	Checklist items 6 and 9 not fulfilled
Succaria,^78^ 2018 (USA)	HNSCC, unspecified	Archived HNSCC specimen	Unspecified	27	Retrospective. IDO expression by tumour cells and infiltrating immune cells	>500 IDO+expressing cells/mm^2^ in 17/27 HNSCC specimens, IDO expressed by tumour cells and infiltrating immune cells in 12/27 (44%) cases (range 5%‐95% tumour cells+)	—
Venkata,^79^ 2017 (India)	HNSCC, unspecified	FFPE	—	50	Retrospective. Stained slides analysed manually and by digital algorithms	Increased expression of IDO in tumour cells correlated with FOXP3‐positive immune cells. Overall percentage of IDO and CD8‐positive immune cells were higher than PD‐L1 and FOXP3‐positive immune cells	—
Seppälä,^35^ 2016 (Finland)	Tongue SCC (58) and lymph node samples (32), control group (30) with tongue squamous cell hyperplasia	FFPE	Anti‐IDO monoclonal antibody, (MAB5412) 10.1, 1:200	108	Retrospective. Semi‐quantitative light microscopic evaluation by two observers. IDO proportion and IDO staining intensity scores were calculated	IDO expression was higher in tongue hyperplasia than SCC. In tumour stage T2‐T4 and tumours with strong inflammation at the invasive front, IDO expression correlated with poor survival	Fulfilled all items
Ye,^36^ 2013 (China)	Laryngeal SCC: Glottic (92), Subglottic (65); Stage: Early I‐II (78), Late III‐IV (109)	FFPE surgical specimen, tissue block with tumour cells and non‐neoplastic laryngeal tissue was selected	IDO, Chemicon (AB5968) 10.1, 1:300	187	Retrospective. IDO staining intensity in the tissue and tumour‐infiltrating lymphocytes. Correlation with survival analysis	Tumour IDO expression not significantly correlated with histology, clinical/nodal stage or tumour differentiation, but positively associated with density of FOXP3+ TILs (*P* = .028). High IDO expression was an independent predictor of poor DFS (HR = 3.973, *P* = .026) and OS (HR = 3.258, *P* = .029)	Fulfilled all items
Kuales,^80^ 2011 (Germany)	Lower lip SCC	Lesional biopsies, FFPE	Anti‐IDO monoclonal antibody, Millipore (10.1), 1:150	47	Retrospective. Density of inflammatory infiltrate at the invasive front of each tumour was calculated	IDO expression correlated with moderate to intense inflammatory infiltrate and was found in myeloid CD11c+ S100+ DCs along the border of invasive tumour cells where Foxp3 regulatory T cells were also present	—
Laimer,^37^ 2011 (Austria)	Oral SCC	Paraffin blocks, deparaffinised and rehydrated sections mounted on slides	Anti‐IDO human antibody, Chemicon, Millipore (ab9252) sheep polyclonal, 1:500	88	Retrospective. IDO expression was evaluated and total expression score given. Cox proportional hazard model for the relationship of IDO expression with survival time	IDO expression, staging, tumour grade 3 were prognostic for poorer overall survival. IDO was a prognostic factor in patients who received adjuvant (radio)chemotherapy, but had no impact in patients without adjuvant therapy	Checklist items 6 and 9 not fulfilled
Ferdinande,^81^ 2008 (Belgium)	Tonsil SCC (26), Tongue SCC (12)	Unspecified	—	38	Inflammatory infiltrate evaluated and scored (semi)quantitatively	73% of tonsil SCC and 92% of tongue SCC showed IDO expression in tumour cells, focally at invasive front, and no association was found with TNM stage. IDO was present mostly in DCs	—

Abbreviations: REMARK, Reporting Recommendations for Tumour Marker Prognostic Studies^77^; SCC, squamous cell carcinoma; FFPE, formalin‐fixed paraffin‐embedded; IHC, immunohistochemistry; TIL, tumour‐infiltrating lymphocytes; DFS, disease‐free survival; DC, dendritic cell.

### 3.4 IDO gene transcription studies in HNSCC

We included 10 studies which investigated IDO gene transcription, summarised in Table 3. A variety of sources including tissue and blood specimen, 3D tumour microspheres, The Cancer Genome Atlas (TCGA) and Gene Expression Omnibus (GEO) were used to acquire transcription data for analysis. Methods of analysis included single‐sample gene set enrichment analysis (ssGSEA), NanoString analysis, MassARRAY, quantitative polymerase chain reaction (qPCR) and gene expression analysis in peripheral blood mononuclear cells (PBMCs). IDO was strongly expressed in human papillomavirus (HPV) positive HNSCCs and correlated with E7 HPV antigen expression.^39^ IDO1 was overexpressed in tumours from never‐smokers and never‐drinkers; and gene expression profiles showed that IDO1 together with PD‐L1 were co‐overexpressed in HNSCCs^40^, ^41^, ^42^ compared to IDO1 presence in normal head and neck tissue.^42^ Recent studies of methylation of CpG sites suggest that IDO1 expression levels are epigenetically regulated by DNA methylation and hypermethylation of IDO1 is associated with poor overall survival.^43^, ^44^ Measuring expression during treatment, a significant (3.6‐fold) increase was seen in IDO expressed in PBMCs during radiotherapy for patients with stage III‐IV HNSCC.^45^ Whereas after chemoradiation treatment, IDO1 mRNA levels correlated with worse overall survival, and a combined decrease in expression of PD‐L1 and IDO1 post‐treatment was associated with better progression‐free and overall survival.^46^

**TABLE 3:** IDO gene transcription studies in HNSCC

First author, year (country)	Cases (n), source	Method of analysis	Results interpretation
Economopoulou,^46^ 2020 (Greece)	113 locally advanced HNSCC patients who underwent cisplatin chemoradiation, peripheral blood collected at baseline and 1 wk after end of treatment	Expression of IDO1 in the EpCAM+CTC fraction before and after cisplatin chemoradiation. Multivariate Cox regression analysis was used to assess the prognostic value of PD‐L1 and IDO1 expression	IDO1 was significantly overexpressed at baseline compared to post‐treatment (*P* = .007). IDO1 mRNA expression at baseline was associated with better PFS (HR = 0.19, *P* = .017). Post‐treatment IDO1 mRNA levels correlated with worse OS (HR = 3.27, *P* = .008). Patients with combined decreased expression of PD‐L1 and IDO1 post‐treatment had better PFS (*P* = .043) and OS (*P* = .021)
Sailer,^44^ 2019 (Germany)	528 HNSCC patients, TCGA; and 138 HNSCC patients as a validation cohort from the University Hospital Bonn	Methylation of 3 CpG sites was correlated with mRNA expression, immune cell infiltration, mutational burden, HPV status and OS	IDO1 methylation and IDO1 mRNA expression were inversely correlated in the promotor and promoter flank region. IDO1 promoter flank hypermethylation was associated with poor OS (*P* < .001). These suggest that IDO1 expression levels are epigenetically regulated by DNA methylation
Lecerf,^42^ 2019 (France)	96 HNSCC patients who underwent primary surgery	Real‐time polymerase chain reaction was used to assess the expression of 46 immune‐related genes	IDO1 (75%) was among the most significantly overexpressed immune‐related genes and had significantly higher mRNA expression level in HNSCC compared to normal head and neck tissue (*P* < .0001)
Chen,^43^ 2019 (China)	167 oral SCC gene expression data set (GSE30784) and 54 oral SCC DNA methylation data set (GSE75537), obtained from the GEO	Correlations between methylation level of CpG sites and OS of oral SCC patients were assessed by univariate Cox regression analysis followed by robust likelihood‐based survival analysis	A two‐CpG‐based prognostic signature for OSCC OS prediction was obtained, which included the sites cg17892178 and cg17378966 that are located in NID2 and IDO1, respectively
Krishna,^39^ 2018 (USA)	119 HNSCC transcriptomes	Epitope mapping from HPV+HNSCC PBMCs using Elispot, flow cytometry immune cell phenotyping, ssGSEA of HPV and immune gene signatures	IDO was strongly expressed in HPV+ vs HPV− HNSCC (*P* = .001). Its expression correlated with E7 HPV antigen expression (*R* ^2^ = 0.84, *P* = .033). Combined inhibition of PD‐1 and IDO‐1 can sensitise HPV+HNSCCs to CD8+ cytotoxic T‐lymphocyte‐mediated cytotoxicity
Page,^82^ 2018 (USA)	3D‐EX platform, 3D tumour microspheres were produced from fresh HNSCC	NanoString analysis for expression of genes including IDO1	Increased expression of IDO1 gene in HNSCC which were responsive to checkpoint inhibitor treatment ex vivo
Foy,^40^ 2017 (France)	212 oral SCC who were NSND, HPV+samples were excluded. 4 cohorts: TCGA, GEO1, GEO2, CLB	Gene expression profiles generated using microarrays and targeted‐RNA sequencing. Functional pathway analysis performed using ssGSEA and STRING	IDO1 was overexpressed in tumours from NSND vs SD (*P* = .0046). Elderly female patients were more common in NSND and harboured less gene mutations (*P* = .0006). PD‐L1 and IDO1 were co‐overexpressed, suggesting a higher potential benefit of combination therapy involving both
Saâda‐Bouzid,^47^ 2017 (France)	36 recurrent metastatic HNSCC treated with anti‐PD‐1 or anti‐PD‐L1 or in combination with a second checkpoint inhibitor	Extraction of blood DNA and genotyped by MassARRAY and multivariate analysis with PFS and OS	A genotype of IDO1 rs3739319 (A/G or A/A) was associated with a longer PFS and OS, (HR = 8.4, *P* = .002) and (HR = 6.2, CI95% 1.5‐25.9, *P* = .01), respectively. Allelic variation of IDO1 rs3739319G>A, implicated in transcription and regulation was associated with longer survival in patients treated with anti‐PD‐1 therapy
Wirth,^41^ 2017 (USA)	Validation cohort of 25 HPV+HNSCC patients	Microarray of 59 immune‐related genes to compare expression profiles in HPV+HNSCCs. qPCR and protein expression assay used in validation cohort	There was a 65‐fold increase in IDO1 in 10 PD‐L1+ vs 5 PD‐L1− HPV+HNSCCs (*P* = .004). IDO1 expression was upregulated and co‐localised in the TME of the validation cohort. IDO1 expression increased and correlated with disease progression in anti‐PD‐1 treated HNSCC patients
Won,^45^ 2017 (USA)	15 patients with stage III‐IV HNSCC, blood specimen collected before, during and after RT	Gene expression analysis in patients’ PBMCs	A 3.6‐fold (*P* = .1) increase seen in IDO expression in patients’ PBMCs during RT along with other protein mediators that promote immune suppression, suggesting RT could induce tolerogenic effects in HNSCC which can be targeted with checkpoint inhibitor therapy

Abbreviations: HPV, human papillomavirus; PBMCs, peripheral blood mononuclear cells; ssGSEA, single‐sample gene set enrichment analysis; SCC, squamous cell carcinoma; TCGA, The Cancer Genome Atlas; GEO, Gene Expression Omnibus; CLB, Centre Léon Bérard cancer centre, Lyon, France; NSND, never‐smokers and never‐drinkers; SD, smokers drinkers; STRING, search tool for the retrieval of interacting genes/proteins; qPCR, quantitative polymerase chain reaction; PFS, progression‐free survival; OS, overall survival; RT, radiotherapy.

### 3.5 Clinical trials of IDO inhibitors in HNSCC

Clinical trials with published results on IDO in HNSCC are all in early phase (I‐II) and assessed IDO1 inhibitor in combination with a PD‐1/PD‐L1 inhibitor (Table 4). All HNSCC patients enrolled in the published trials had advanced metastatic or recurrent disease. Pembrolizumab^12^, ^14^, ^15^, ^17^ (PD‐1 inhibitor) was most commonly combined with IDO inhibitors, followed by Nivolumab^16^ (PD‐1 inhibitor), Durvalumab^13^ and Atezolizumab^11^ (both PD‐L1 inhibitors). The trials showed (a) objective responses (34%‐55%) and disease control rates (62%‐70%) for Epacadostat (IDO1 inhibitor) in combination with a PD‐1 inhibitor,^12^, ^14^, ^16^ (b) safety profile of IDO1 and PD‐L1 inhibitor combination therapy was consistent with previous reports of each checkpoint inhibitor as monotherapies^13^ and (c) IDO gene expression as a predictive biomarker for response to PD‐L1 therapy.^18^ The most common treatment‐related adverse event associated with the immune checkpoint trials was fatigue (22%‐32%).^11^, ^13^, ^15^ Existing clinical trials testing IDO inhibitors in HNSCC, registered on ClinicalTrials.gov without published results are summarised in Table S2.

**TABLE 4:** Clinical trials of IDO inhibitors in HNSCC with published results

First author, year (country)	Trial name ID	Phase	Design	Disease	Eligibility	Target(s)	Treatments	Patients (n)	Primary end point	Status	Results
Jung,^11^ 2019 (South Korea)	NCT02471846	I	Open‐label, multicentre, dose‐escalation and expansion trial	Locally advanced, recurrent or metastatic incurable solid malignancy	Progression following at least one standard therapy	IDO1 PD‐L1	Navoximod Atezolizumab	157 total 6 HNSCC	Percentage of participants with DLTs and AEs	Completed	75% experienced TRAEs; most common were fatigue (22%) and rash (22%). This combination demonstrated acceptable safety profile
Mitchell,^12^ 2018 (USA)	ECHO‐202/KEYNOTE‐037 NCT02178722	I/II	Multicentre, non‐randomised, open‐label trial	Advanced solid tumours: stage IIIB, IV or recurrent NSCLC, melanoma, RCC, EA, UC, TNBC or HNSCC	All patients with one or more previous therapy, or no available curative treatment	IDO1 PD‐1	Epacadostat Pembrolizumab	62 total 2 HNSCC	Number of subjects with DLTs, ORR	Active, not recruiting	84% experienced TRAEs, none led to death. ORR = 55% (12/22). In HNSCC, 1 CR, 1 SD. Combined safety profile similar to Pembrolizumab monotherapy
Naing,^13^ 2018 (USA)	ECHO‐203 NCT02318277	I/II	Dose‐escalation, open‐label trial	Advanced solid tumours: PC, melanoma, NSCLC, HNSCC	Failed at least 1 prior treatment, intolerant to or refused standard treatment	IDO1 PD‐L1	Epacadostat Durvalumab	34 total	Incidence of DLTs, ORR	Active, not recruiting	Most common TRAE was fatigue (32%), no TRAEs led to death. Safety profile consistent with each as monotherapy
Hamid,^14^ 2017 (USA)	ECHO‐202/KEYNOTE‐037 NCT02178722	I/II	Multicentre, non‐randomised, open‐label trial Reporting of HNSCC results	HNSCC	Metastatic HNSCC with ≥1 prior CT regimen	IDO1 PD‐1	Epacadostat Pembrolizumab	38 HNSCC	Number of subjects with DLTs, ORR	Active, not recruiting	ORR 34% (2 CR, 8 PR), DCR 62% (8 SD) in patients with 1‐2 prior therapies. Response observed regardless of HPV status
Hamid,^15^ 2017 (USA)	ECHO‐202/KEYNOTE‐037 NCT02178722	II	Multicentre, non‐randomised, open‐label trial Reporting of phase II safety	Advanced or recurrent NSCLC, melanoma, RCC, EA, UC, TNBC or HNSCC	All patients with one or more previous therapy, or no available curative treatment	IDO1 PD‐1	Epacadostat Pembrolizumab	244 total	Number of subjects with DLTs, ORR	Active, not recruiting	55% discontinued treatment, mainly due to disease progression (n = 97). Main TRAE was fatigue (23%)
Perez,^16^ 2017 (USA)	ECHO‐204 NCT02327078	I/II	Non‐randomised, open‐label trial	Advanced cancers: melanoma, NCSCLC, HNSCC, CRC, OVC, GBM, B‐cell NHL	All adult patients with pathologically confirmed disease	IDO1 PD‐1	Epacadostat Nivolumab	241 total 36 phase I 205 phase II 23 HNSCC	Phase I: safety and tolerability with DLTs Phase II: ORR, PFS, OS	Active, not recruiting	Most common TRAEs: rash, fatigue, nausea, no treatment‐related deaths, 70% DCR in HNSCC
Gangadhar,^17^ 2016 (USA)	ECHO‐202/KEYNOTE‐037 NCT02178722	I	Multicentre, non‐randomised, open‐label trial	Advanced melanoma and select solid tumours	All patients with one or more previous therapy, or no available curative treatment	IDO1 PD‐1	Epacadostat Pembrolizumab	62 total 2 HNSCC	Number of subjects with DLTs, ORR	Active, not recruiting	Reponses in 2 patients with HNSCC; 1 PR, 1 SD
Seiwert,^18^ 2016 (USA)	KEYNOTE‐012 NCT01848834	Ib	Non‐randomised, open‐label, multicentre trial	PD‐L1 positive recurrent or metastatic HNSCC	Pathologically confirmed disease, any number of prior treatment regimens	PD‐1	Pembrolizumab	60 total 23 HPV(+) 37 HPV(−)	Incidence of AEs, number discontinuing due to AEs, ORR	Active, not recruiting	IDO1 found as part of six interferon‐γ related gene signature. Responders had higher IDO1 expression *P* = .039

Abbreviations: NSCLC, non‐small‐cell lung cancer; RCC, renal cell cancer; EA, endometrial adenocarcinoma; UC, urothelial carcinoma; TNBC, triple‐negative breast cancer; DLT, dose‐limiting toxicities; ORR, overall response rate; TRAE, treatment‐related adverse event; CR, complete response; PR, partial response; SD, stable disease; PC, pancreatic cancer; CT, chemotherapy; DCR, disease control rate; CRC, colorectal cancer; OVC, ovarian cancer; GBM, glioblastoma; NHL, non‐Hodgkin lymphoma; PFS, progression‐free survival; OS, overall survival; AE, adverse event.

## 4 DISCUSSION

### 4.1 Summary of main results

Existing evidence suggests that IDO is integral to TME immunity in HNSCC in the context of positive HPV status, modulating existing therapies and application in combinatorial immunotherapy. The differential expression of IDO and predicted response to immunotherapy based on simulation models as shown by cell line studies suggests that differences in IDO as a biomarker may be related to the stage of disease, site of tumour, tissue type or genomic differences between patients. These findings further support a personalised approach to future HNSCC therapies. To date, only four retrospective studies have investigated IDO expression in formalin‐fixed paraffin‐embedded tumour specimen as a prognostic biomarker for HNSCC.^35^, ^36^, ^37^, ^38^ Interestingly, Laimer^37^ and colleagues showed that IDO was a prognostic factor in patients who received adjuvant (radio)chemotherapy, but had no impact in patients without adjuvant therapy. Saâda‐Bouzid^47^ showed that IDO1 rs3739319 (A/G or A/A) was associated with a longer progression‐free survival and overall survival. They suggest that the prognostic and predictive value of IDO polymorphism should be tested prospectively in the context of immune checkpoint inhibitor era. Wirth and colleagues^41^ showed an increase in IDO1 in PD‐L1+ HPV+HNSCCs and proposed that IDO1 is an adaptive immune resistance pathway to anti‐PD‐1 monotherapy. These results support the rationale for future combinatorial therapies involving IDO1 and PD‐1. Liang et al^30^ showed that STING activation, as indicated by staining, was greatest around the nucleus in a majority (16 of 25) of HPV‐positive tongue SCC samples and was present in the whole cytoplasm in 22 of 25 HPV‐negative samples. They suggest that HPV hijacks and activates STING by DNA sensing which induces an immunosuppressive microenvironment through IDO expression and recruitment of regulatory T cells allowing the establishment of tumourigenesis in tongue SCC. The central role of IDO expression and activation by STING is also consistent with previous work on DNA sensing via STING by Huang,^48^ Lemos^49^ and colleagues.

### 4.2 IDO inhibitors commonly applied in pre‐clinical and clinical studies

IDO can be expressed in tumour cells but multiple TME cell types may also express IDO including dendritic cells, macrophages, fibroblasts, endothelial/epithelial cells and PBMCs,^50^, ^51^ though lymphoid cells (eg T cells and tumour‐infiltrating lymphocytes) rarely express IDO. It is important to distinguish between IDO protein abundance in the TME (detected by IHC) and IDO enzyme activity (measured peripherally in the blood and in situ using homogenised tissues), as multiple factors impact IDO activity including enzyme cofactors and natural inhibitors such as hemin and nitric oxide, respectively. As IDO inhibits innate and adaptive immunity, IDO inhibitors (IDOi) have been tested as drugs to potentiate antitumour immunity in the TME. A recent review by Lemos and colleagues summarised the IDOi under pre‐clinical and clinical evaluation.^52^ In total there are seven IDOi drugs under evaluation in Phase I‐III clinical trials and four that are applied in pre‐clinical studies. The two front‐running drugs in development are the non‐selective IDOi indoximod (also known as D‐1MT or NLG‐8186) and the tryptophan competitive IDOi epacadostat (INCB024360). Drugs being tested in clinical trials include the non‐selective IDO and TDO (tryptophan 2,3‐dioxygenase) inhibitor navoximod (NLG‐919),^53^, ^54^ which is approximately tenfold more selective for IDO1 than TDO2; the selective IDO1 inhibitor linrodostat (BMS‐986205)^55^ and IDO1 and TDO2 inhibitor PF‐06840003^56^ which are both more than 100‐fold selective for IDO1 than TDO2. Indoximod, epacadostat and linrodostat are also being evaluated in combination drug trials given the strong rationale for the use of IDOi drugs as immunometabolic adjuvants to increase the efficacy of (chemo)radiotherapy and immunotherapies.^57^ In vitro studies of IDOi drugs in cancer include those investigating indoximod^58^ and linrodostat.^59^ Applied as monotherapy to patient‐derived colorectal cancer cell lines, indoximod exhibited rather low direct cytotoxic activity, whereas coculturing the cell lines in an allogeneic setting using naïve, “unprimed” lymphocytes from healthy volunteers generally boosted the antitumoural effect of indoximod.^58^ However, this was not tested in an autologous setting using partially exhausted lymphocytes from cancer patients. Interestingly, low IDO expressing cells responded better to indoximod monotherapy, suggesting that indoximod likely targets additional pathways, although the precise mechanism of action is yet to be elucidated. It is important to stress that IDOi drugs may promote antitumour effects by targeting tumour accessory cells in malignant lesions and/or in tumour‐draining lymphoid tissues, and not necessarily by targeting tumour cells per se. Nonetheless, multiple studies support the use of non‐selective IDOi drugs to counteract tumour‐induced immunosuppression and increase antitumour efficacy in combination with other therapeutics.

### 4.3 Other combination immunotherapeutic strategies

**FIGURE 2:**
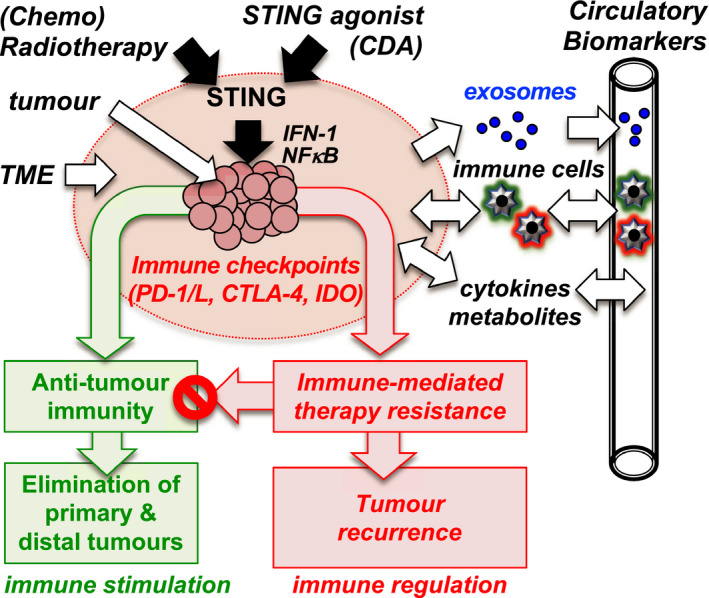
IDO immune microenvironment hypothesis. (Chemo)radiotherapy and CDA treatments activate STING to incite antitumour immunity but also boost immune regulation to enhance therapy resistance. Multiple STING‐responsive pathways involving chronic inflammation and tumour progression and associated with immune checkpoints (PD‐1/L, CTLA‐4, IDO) result in therapy resistance. Blocking these pathways modulate the TME in favour of antitumour immunity. Immune, inflammatory and metabolic biomarkers in blood reflect changes in the TME caused by treatments and therapy resistance. Abbreviations: CDA, cyclic diadenyl monophosphate; PD‐1, programmed cell death protein 1; PD‐L1, programmed death ligand 1; CTLA‐4, cytotoxic T‐lymphocyte‐associated antigen 4; IDO, indoleamine 2,3‐dioxygenase; IFN‐1, interferon‐1; NFkB, nuclear factor kappa‐light‐chain‐enhancer of activated B cells

Apart from immune checkpoint blockade alone, other combination immunotherapeutic strategies have been studied. Rational combinations have been tested based on the knowledge that activating STING in the TME of mice stimulated protective antitumour immunity; however, preliminary outcomes from a clinical trial reveal little benefit of STING agonist monotherapy.^60^ To overcome this therapy resistance, Lemos and colleagues showed that in mice bearing established Lewis lung carcinoma (LLC) tumours, intratumoural treatment with STING agonist, synthetic cyclic diadenyl monophosphate (CDA) and co‐treatment with selective COX2 inhibitor celecoxib eliminated the primary tumour burden, prevented metastases and induced durable protective antitumour immunity.^61^ Co‐treatment with IDOi drugs indoximod, navoximod and linrodostat also enhanced antitumour responses to CDA, especially in co‐treatment with linrodostat which induced rapid tumour regression and increased survival, however, did not eliminate the primary tumours. Interestingly, inhibiting COX2 also significantly reduced IDO activity, which may contribute to greater antitumour activity elicited by celecoxib in combination with CDA. Another strategy to enhance antitumour immunity is to deliver recombinant enzymes that act downstream of IDO to reduce the level of immune‐suppressive Trp catabolites in the TME. PEGylated Kynureninase (KYNU) combined with immune checkpoint inhibitors or cancer vaccine reduced Kyn levels in the TME, attenuated immune suppression and promoted tumour control in vivo^62^. However, the effectiveness of this strategy in promoting clinical responses in patients is yet to be proven. Although speculative at present, combining IDOi drugs with radiotherapy treatment may modulate the TME in favour of antitumour activity and help overcome treatment resistance in cases where the disease is less radiosensitive, for instance in HPV‐negative HNSCCs where radiotherapy controls less than 50% of disease cases that have concurrent nodal metastases. The hypothesised role of IDO in the immune microenvironment is summarised in Figure 2.

### 4.4 Strengths, limitations and potential bias of evidence

This narrative systematic review synthesises the existing evidence on studies involving IDO in HNSCC. Due to the heterogeneity among the studies, a meta‐analysis was not possible and a qualitative analysis was therefore performed on the groupings of study types. Although existing HNSCC cell line studies showed IDO involvement in certain cell lines (SCC4, SCC15) and influenced by STING activation, conclusions cannot be drawn from these studies on the influence of the whole immune system or local TME dendritic cell response on the IDO pathway. Considering current published clinical trials evidence, all trials have tested an IDO1 inhibitor in combination with either a PD‐1 or PD‐L1 inhibitor. Potential reasons for a limited response seen with IDO inhibitors in existing trials could be (a) poor IDO inhibitor with a short half‐life, (b) ineffective dosing regimen and (c) redundant mechanisms and/or no synergy between PD‐1/PD‐L1 and IDO pathways. Furthermore, there has been no report of IDO immune‐based therapy or IDO inhibitor therapy in comparison with current standard of care treatments for HNSCC. In addition to IDO there are also other enzymes which have an influence on tryptophan metabolism. Apart from IDO, tryptophan 2,3‐dioxygenase (TDO2) is another rate‐limiting enzyme of the kynurenine pathway (KP).^63^ TDO2 is mainly expressed in the liver; however, it has also been shown to be overexpressed in tumours as a means of immune evasion.^64^, ^65^, ^66^ Although TDO2 and IDO activity cannot be distinguished based on peripheral blood analysis of KP activity, Riess et al^33^ showed that glioblastoma multiforme (GBM) tumours had higher TDO2 expression whilst HNSCC tumours mainly presented with IDO1. While IDO is responsive to inflammatory signals (eg interferons), stress‐related signals such as glucocorticoids induce TDO2 expression, highlighting the radical differences in upstream pathways that induce KP metabolic activity. In their series an HPV‐positive HNSCC case showed the highest abundance of IDO1, reflecting its higher relative immunogenicity. In the current literature, there exists sparse evidence of the influence of TDO2 in HNSCC; however, it is possible for TDO to have an effect on the immune TME given its role in metabolic KP activity.

### 4.5 Implications for future clinical practice and research

**FIGURE 3:**
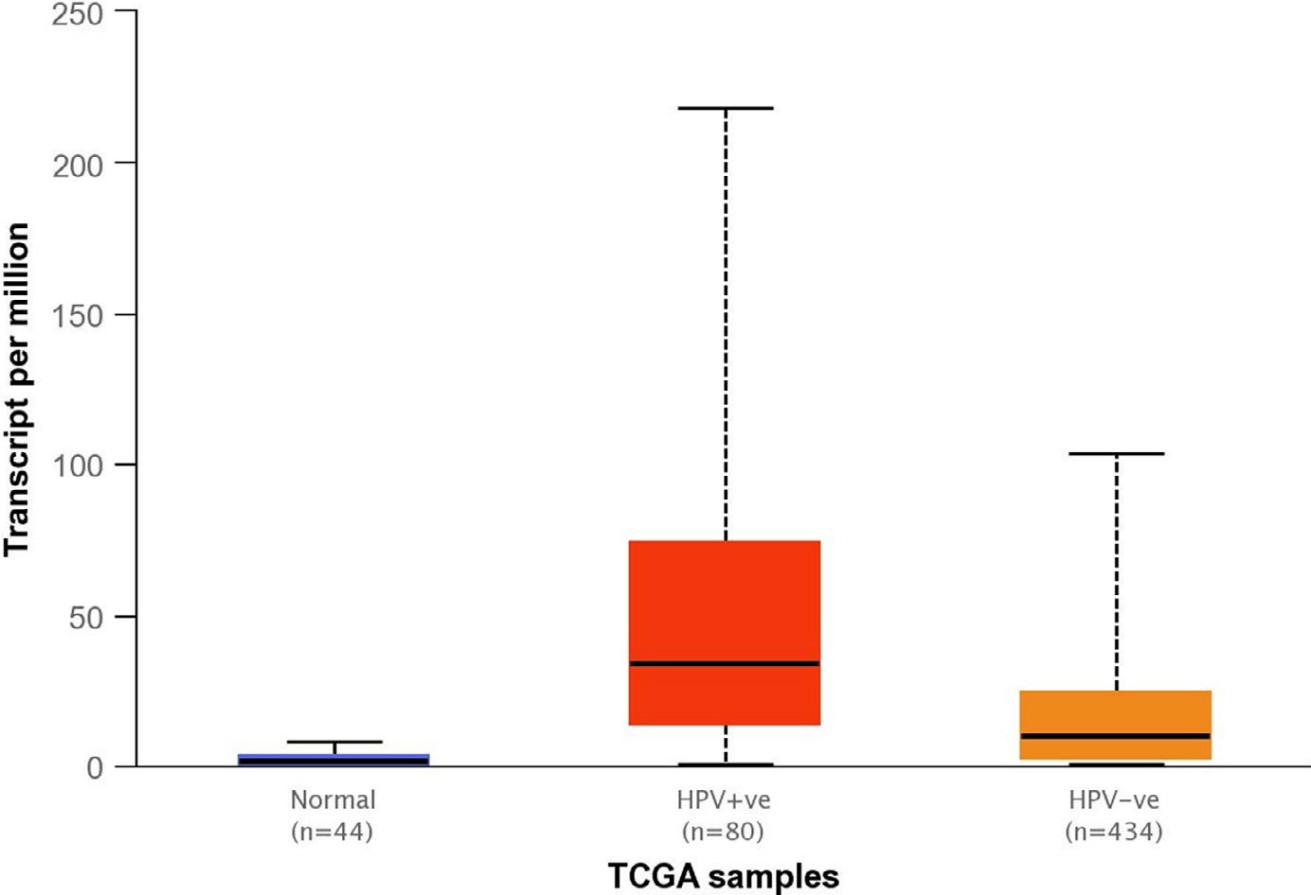
Expression of IDO1 in HNSCC based on HPV status. Box and whisker plots of IDO1 expression in HPV‐positive, HPV‐negative and normal adjacent tissue generated from TCGA data. This comparison shows significant differences in IDO1 expression when comparing: HPV‐positive vs normal tissue (*P* = .00002), HPV‐positive vs HPV‐negative (*P* = .00112) and HPV‐negative vs normal tissue (*P* < .00001)

Although there exists a spectrum of immune cell infiltrates in HNSCC, it is now recognised that HPV‐positive and HPV‐negative HNSCCs have distinctly different immunophenotypes characterised by increased T‐cell infiltrate, more immune cells expressing PD‐L1 and increased presence of markers of immune activation (eg granzyme and perforin) in HPV‐positive tumours whilst HPV‐negative tumours tend to have less abundant immune cell infiltrates including T cells and Tregs.^67^ The immunophenotype of HNSCCs including the presence of immune cell infiltration and immune checkpoints within the TME of HPV‐positive and HPV‐negative tumour types may be important predictive biomarkers that will help guide personalised immunotherapeutic approaches in the future. A comparison of IDO1 expression data from TCGA shows that HPV‐positive HNSCCs have significantly higher IDO1 expression when compared to HPV‐negative HNSCCs (*P* = .001) and adjacent normal tissue (*P* < .001) (Figure 3)^68^ although the IDO expression is also elevated in HPV‐negative HNSCC when compared to normal tissue. Furthermore, studies in various solid cancers investigating the differences between primary tumour and corresponding lymph node metastases have shown that strong tumoural IDO expression is associated with metastatic disease.^69^, ^70^, ^71^ It has been observed in both colorectal and breast cancer that IDO expression pattern is consistent between the primary tumour and metastatic sites.^72^, ^73^ This suggests IDO expression as a modulator of cancer inflammation and immune evasion in both primary and metastatic tumour progression.^51^ As more evidence mounts in favour of the involvement of IDO in the TME of HNSCC and a better understanding of its mechanistic role in HNSCC immune modulation emerges, IDO‐based therapies are likely to be translated to clinical practice to improve outcomes for HNSCC patients. An increasing number of new IDO inhibitors are currently being discovered. An example is DN‐016 a highly potent, selective, orally available IDO1 inhibitor with good absorption, distribution, metabolism and excretion and safety profile which was presented at ASCO 2018.^10^ Also, HTI 1090 a dual inhibitor of IDO1 and hepatic enzyme tryptophan 2,3‐dioxygenase (TDO) is currently in phase I trial for advanced solid tumours including HNSCC.^74^ Considered “best in class” IDO1 inhibitor, BMS‐986205 was recently halted from phase III trial^75^ but earlier‐phase combination studies are still ongoing. IDO inhibitor utility as a single agent or in combination with other checkpoint inhibitors is still under clinical evaluation and recently published results show promising overall response rates in HNSCC.^12^ Existing evidence suggests an increasing role for IDO‐based therapies in the context of PD‐L1 or HPV‐positive HNSCCs; however, this observation requires more mechanistic evidence to elucidate the relationship.

Future research on IDO in HNSCC should seek to address the following questions:

1. Is the IDO pathway regulated by treatment (eg radiotherapy)?
2. Can IDO immune status be used as a predictive biomarker of treatment outcome?
3. What is the relationship between the IDO pathway and HPV or PD‐L1 status in HNSCC?

Improved understanding of the mechanistic role of IDO in modulating local TME immunity will inform the targeting of the IDO pathway to optimise HNSCC therapy. The ability to measure IDO activity in the peripheral blood of cancer patients^76^ allows researchers to prospectively map and characterise potential groups of patients who may benefit from modified treatment doses (eg radiotherapy) based on IDO immune status. The correlation of IDO activity and expression at the tumour, draining lymph nodes, and in peripheral blood can potentially lead to less invasive sentinel lymph node and liquid biopsies to inform stratified, personalised HNSCC immune‐based therapy.

## 5 CONCLUSIONS

Current evidence shows the presence of IDO in the TME and suggests a link to prognosis and prediction of HNSCC treatment outcome. However, the exact mechanism of immune modulation by the IDO pathway in the TME of HNSCC remains unclear. Future translational studies need to prospectively map the activity and expression of IDO throughout HNSCC treatment to achieve a mechanistic understanding of its involvement in TME immunity and to inform the design of precision, stratified immunotherapeutic approaches involving IDO.

## CONFLICT OF INTEREST

The authors have no conflict of interest to declare.

## Supporting information

Table S1‐S2

## Data Availability

Data sharing is not applicable to this article as no new data were created or analysed in this study.
