# Supplementary material for: The immunotherapeutic role of indoleamine 2,3‐dioxygenase in head and neck squamous cell carcinoma: A systematic review
Source: Clin Otolaryngol. 2021 May 30;46(5):919–34. doi: 10.1111/coa.13794 (PMC8600953; doi:10.1111/coa.13794)
Supplement: Supplementary file 1 — Table S1‐S2 [file COA-46-919-s001.docx]

**Supplementary tables**

**Supplementary table S1. Included conference abstracts**

| **First author (Country)** | **Year** | **Conference** | **Journal** | **Results interpretation** | **Type of study** |
| --- | --- | --- | --- | --- | --- |
| Naing^12^ (USA) | 2018 | AACR 2018 | Cancer Research | Epacadostat (IDO-1 inhibitor) plus durvalumab (PD-L1 inhibitor) in advanced cancer was well tolerated; safety profile consistent with each as monotherapy. Epacadostat 100 and 300 mg BD are being evaluated in Phase 2 expansions with HNSCC. | Clinical trial, Phase I/II |
| Page^49^ (USA) | 2018 | AACR 2018 | Cancer Research | In tumour samples (including HNSCC) responsive to checkpoint inhibitor treatment ex vivo, Nanostring analysis revealed increased expression of IDO1 gene. | IDO gene transcription |
| Subramanian^29^ (USA) | 2018 | AACR 2018 | Cancer Research | High levels of Kyn in HNSCC cell lines shown through metabolic profiling via MS. Checkpoint inhibition of IDO1 leads to increased apoptosis in vitro. | Cell line |
| Succaria^45^ (USA) | 2018 | AACR 2018 | Cancer Research | >500 IDO+ expressing cells/mm2 in 17/27 HNSCC specimens, IDO expressed by tumour cells and infiltrating immune cells in 12/27 (44%) cases (range 5-95% tumour cells+). | Tumour IHC |
| Hamid^13^ (USA) | 2017 | ASCO 2017 | J Clin Oncology | Patients with 1-2 therapy prior to epacadostat and pembrolizumab had a 34% ORR (2 CR, 8 PR) and a 62% DCR (8 SD), regardless of HPV status. | Clinical trial, Phase I/II |
| Hamid^14^ (USA) | 2017 | ASCO 2017 | J Clin Oncology | Epacadostat 100 mg BD plus pembrolizumab 200 mg Q3W showed acceptable safety profile, therefore supports continued evaluation of combination therapy. | Clinical trial, Phase II |
| Perez^15^ (USA) | 2017 | ASCO 2017 | J Clin Oncology | Preliminary DCR was 70% (n=16) of the 23 enrolled, efficacy-evaluable patients with HNSCC treated with epacadostat 300 mg. | Clinical trial, Phase I/II |
| Saâda-Bouzid^38^ (France) | 2017 | MAP 2017 | Annals of Oncology | In recurrent or metastatic HNSCC treated with anti-PD1 based treatment, allelic variation of IDO1 rs3739319G>A was significantly associated with longer PFS (p=0.04). | IDO gene transcription |
| Venkata^46^ (India) | 2017 | ESMO 2017 | Annals of Oncology | A positive correlation was seen in the expression of IDO in the tumour cells as the overall percentage of IDO and CD8 positive immune cells increased compared to PDL1 and FOXP3 positive immune cells. | Tumour IHC |
| Wirth^36^ (USA) | 2017 | ASCO 2017 | J Clin Oncology | IDO1 expression increased 65-fold in 10 PD-L1(+) as compared to 5 PD-L1(-) HPV(+) HNSCC (p=0.004). PD-L1 and IDO1 co-localised within the TME of HPV(+) HNSCC patients. In anti-PD1 therapy, IDO1 expression increased two-fold and correlated with disease progression. | IDO gene transcription |
| Won^37^ (USA) | 2017 | AACR-AHNS 2017 | Clin Cancer Research | IDO gene expression was increased 3.6-fold in PBMC of patients, along with IL-1beta and VEGF during radiotherapy. | IDO gene transcription |
| Gangadhar^16^ (USA) | 2016 | ESMO 2016 | Annals of Oncology | Reponses were seen in 2 patients with HNSCC; 1 PR, 1 SD. | Clinical trial, Phase I |
| Ferdinande^48^ (Belgium) | 2008 | IAP 2008 | Histopathology | 73% of tonsil SCC and 92% of tongue SCC showed IDO expression in tumour cells, and no association was found with TNM classification. | Tumour IHC |

Abbreviations: AACR = American Association for Cancer Research, ASCO = American Society of Clinical Oncology, MAP = Molecular Analysis for Personalised Therapy, ESMO = European Society for Medical Oncology, AHNS = American Head and Neck Society, ASTRO = American Society for Radiation Oncology, CARO = Canadian Association of Radiation Oncology, IAP = International Academy of Pathology, MS = mass spectrometry, ORR = overall response rate, CR = complete response, PR = partial response, DCR = disease control rate, SD = stable disease, PD-L1 = programmed death-ligand 1, HPV = human papillomavirus, qPCR = quantitative PCR, PBMC = peripheral blood mononuclear cells.

**Supplementary table S2. Clinical trials of IDO inhibitors in HNSCC without published results**

| **First author, year (Country)** | **Trial name**  **ID** | **Phase** | **Design** | **Disease** | **Eligibility** | **Target(s)** | **Treatments** | **Enrolment (n)** | **Primary end point** | **Status** |
| --- | --- | --- | --- | --- | --- | --- | --- | --- | --- | --- |
| Atridia Pty Ltd.^41^, 2017 (Australia) | A Trial of HTI-1090 in Subjects With Advanced Solid Tumors  NCT03208959 | I | Open-label, multicentre, non-randomised, dose-escalation trial | Advanced solid tumours | Adults with advanced or metastatic disease refractory to or relapsed from standard therapies | IDO1 | HTI-1090 | 30 (estimated) | Adverse events, laboratory result/vital sign/ECG abnormalities | Recruiting |
| Bristol-Myers Squibb^42^, 2017 (USA) | An Immuno-therapy Study of Nivolumab in Combination With Experimental Medication BMS-986205 Compared to Standard of Care EXTREME Regimen in First-line Recurrent/Metastatic Squamous Cell Carcinoma of Head and Neck  NCT03386838 | III | Randomised, global, open-label trial | HNSCC (oral cavity, oropharynx, hypopharynx and larynx) | Adults with recurrent or metastatic disease that is not amenable to therapy with curative intent | IDO1  PD-1 | BMS-986205  Nivolumab | 1 (actual) | PFS, OS, ORR, number of (serious) adverse events, TTSD | Terminated |
| Eli Lilly and Company^50^, 2017 (USA) | A Study of LY3381916 Alone or in Combination With LY3300054 in Participants With Solid Tumors  NCT03343613 | Ia/Ib | Non-randomised, open-label | HNSCC, NSCLC, UC, brain metastasis | Adults with advanced and/or metastatic cancer | IDO-1  PD-L1 | LY3381916  LY3300054 | 290 (estimated) | DLTs, PKs, ORR, TTR, DCR, DOR, PFS | Recruiting |
| Hayreh^51^, 2017 (USA) | Nivolumab Plus Epacadostat in Combination With Chemotherapy Versus the EXTREME Regimen in Squamous Cell Carcinoma of the Head and Neck (CheckMate 9NA/ECHO-310)  NCT-03342352 | III | Randomised, global trial | HNSCC (oral cavity, oropharynx, hypopharynx and larynx) | Adults with recurrent/metastatic disease | IDO-1  PD-1 | Epacadostat  Nivolumab  Placebo  Carboplatin  Cisplatin  Cetuximab  5-Fluorouracil | 0 | PFS, OS, ORR, DOR, TTSD | Withdrawn |
| Jones^52^, 2017 (USA) | Pembrolizumab Plus Epacadostat, Pembrolizumab Monotherapy, and the EXTREME Regimen in Recurrent or Metastatic Head and Neck Squamous Cell Carcinoma (KEYNOTE-669/ECHO-304)  NCT03358472 | III | Randomised, open-label trial | HNSCC | Adults who have had pre-treatment tumour biopsy | IDO1  PD-1 | Epacadostat  Pembrolizumab  Cetuximab + Cisplatin or  Carboplatin +  5-Fluorouracil | 89 (actual) | ORR and safety and tolerability | Active, not recruiting |
| Seiwert^53^, 2017 (USA) | Neoadjuvant Pembrolizumab + Epacadostat Prior to Curative Surgical Care for Squamous Cell Carcinoma of the Head and Neck: The KEO Trial  NCT03325465 | II | Single arm, open-label trial | HNSCC | Adults with non-bulky HNSCC with an indication for surgical therapy | IDO1  PD-1 | Epacadostat  Pembrolizumab | 44 (estimated) | Rate of MTE and CR. PFS, OS and adverse events | Not yet recruiting |
| Zheng^54^, 2017 (USA) | A Study of Epacadostat in Combination With Pembrolizumab and Chemotherapy in Subjects With Advanced or Metastatic Solid Tumors (ECHO-207/KEYNOTE-723)  NCT03085914 | I/II | Non-randomised, open-label | Advanced or metastatic solid tumours | Adults with advanced or metastatic solid tumours | IDO1  PD-1 | Epacadostat  Pembrolizumab | 70 (actual) | Safety and tolerability, ORR, DOR | Active, not recruiting |

Abbreviations: MTE = major treatment effect, CR = complete response, PFS = progression-free survival, OS = overall survival, ORR = objective response rate, DLT = dose limiting toxicities, PK = pharmacokinetics, TTR = time to response, DCR = disease control rate, DOR = duration of response, TTSD = time to meaningful symptomatic deterioration, ECG = electrocardiogram.
